# Correction: Eroglu et al. Biodistribution of Tc-99m-Labeled Solid Lipid Nanoparticles and Evaluation of Their Possibility as a Radiopharmaceutical. *Molecules* 2026, *31*, 654

**DOI:** 10.3390/molecules31111909

**Published:** 2026-06-02

**Authors:** Hayrettin Eroglu, Arif Kürsad Ayan, Ayse Yenilmez

**Affiliations:** 1 Department of Chemical Engineering, Faculty of Engineering and Architecture, Erzurum Technical University, 25110 Erzurum, Türkiye; 2 Department of Nuclear Medicine, Samsun Medicana Hospital, 55100 Samsun, Türkiye; ayankursad@gmail.com; 3 Department of Mathematics and Science Education, Faculty of Education, Van Yüzüncü Yıl University, 65080 Van, Türkiye; ayseyenilmez@yyu.edu.tr

There was an error in the original publication [1], the Institutional Review Board Statement is missing. The Institutional Review Board Statement should be added as below:

The animal study was conducted in accordance with the relevant institutional guidelines and ethical standards, and the protocol was approved by the Atatürk University Animal Experiments Local Ethics Committee (Approval No. 83443830/891) on 27 February 2015.

The authors state that the scientific conclusions are unaffected. This correction was approved by the Academic Editor. The original publication has also been updated.
